# Endoplasmic Reticulum Stress Inducer Tunicamycin Alters Hepatic Energy Homeostasis in Mice

**DOI:** 10.3390/ijms18081710

**Published:** 2017-08-04

**Authors:** Bin Feng, Xiaohua Huang, Dandan Jiang, Lun Hua, Yong Zhuo, De Wu

**Affiliations:** 1 Animal Nutrition Institute, Sichuan Agricultural University, Chengdu 611130, China; hxh3028@163.com (X.H.); dandanjiang@163.com (D.J.); hualun0516@163.com (L.H.); zhuoyong2003@163.com (Y.Z.); 2 Key Laboratory of Animal Disease-Resistant Nutrition of Ministry of Education, Sichuan Agricultural University, Chengdu 611130, China

**Keywords:** tunicamycin, liver, triglyceride, lipoprotein, glycogen, blood glucose, ER stress, Akt

## Abstract

Disorders of hepatic energy metabolism, which can be regulated by endoplasmic reticulum (ER) stress, lead to metabolic diseases such as hepatic steatosis and hypoglycemia. Tunicamycin, a pharmacological ER stress inducer, is used to develop an anti-cancer drug. However, the effects of tunicamycin on hepatic energy metabolism have not been well elucidated. Mice were intraperitoneally injected with tunicamycin or vehicle. Twenty-four hours later, hepatic triglyceride and glycogen content and serum lipids profiles were analyzed, as well as the expression of lipogenic and gluconeogenic genes. Tunicamycin significantly induced hepatic a yellowish color and ER stress, as well as increasing serum levels of aspartate transaminase and alanine transaminase. Besides, tunicamycin remarkably increased hepatic triglyceride content and suppressed the expression of apolipoprotein B100. In addition, tunicamycin-treated mice had lower serum levels of triglyceride, apolipoprotein B, low-density lipoprotein cholesterol and high-density lipoprotein cholesterol. Gene expression of peroxisome proliferator-activated receptor α was decreased by tunicamycin, but the protein level was increased. Furthermore, blood glucose level and hepatic glycogen content were decreased in tunicamycin-treated mice. Protein kinase B signaling was attenuated in the tunicamycin-treated liver, but the expression and activities of phosphoenolpyruvate carboxykinase and glucose-6-phosphatase were unchanged. Tunicamycin alters hepatic energy homeostasis by increasing triglyceride accumulation and decreasing glycogen content.

## 1. Introduction

Liver, the primary metabolic organ, plays an important role in the systemic energy homeostasis, including glucose production, lipogenesis, fatty acid oxidation, lipoprotein secretion and glycogen synthesis [1]. To be specific, firstly, the liver regulates blood glucose homeostasis by stimulating gluconeogenesis, a process mediated by phosphoenolpyruvate carboxykinase (PEPCK) and glucose 6-phosphatase (G6pase), whose expression are mainly regulated by insulin signaling and endoplasmic reticulum (ER) stress [2,3,4]. Secondly, the liver is one of the leading organs for lipids metabolism, including lipogenesis, cholesterol synthesis and fatty acid oxidation [5]. In the liver, lipogenesis is regulated by enzymes like fatty acid synthase (FAS) and stearoyl-CoA desaturase 1 (SCD1), while fatty acid oxidation is controlled by enzymes such as carnitine palmitoyltransferase 1a (CPT1a) and acyl-CoA dehydrogenase, long chain (ACADL) [6,7]. Stimulation of lipogenesis or inhibition of fatty acid oxidation in the liver leads to hepatic triglyceride accumulation, which results in hepatic steatosis [8]. In addition, reduced apolipoprotein expression and impaired hepatic lipoprotein secretion give rise to liver triglyceride accumulation [9]. Thirdly, liver synthesizes and stores glycogen, which can be broken down at fasting state to provide glucose to keep blood glucose homeostasis [10]. Synthesis of glycogen is regulated by enzyme of glycogen synthetase (GS) while glycogen breakdown is mediated by glycogen phosphotase (GP), both of which are regulated by insulin signaling [10,11]. Moreover, impaired liver glycogen accumulation leads to hypoglycemia under fasting state [10].

In the liver, ER stress restores ER homeostasis by stimulating the activation of inositol requiring enzyme 1 (IRE1), activating transcription factor 6 (ATF6) and RNA-dependent protein kinase-like ER kinase (PERK), which also regulate energy metabolism [4]. Firstly, IRE1 activation prevents the inhibition of insulin signaling on forkhead box O1 (FOXO1) activation, thus induces gluconeogenesis, which is known as insulin resistance [4]. Secondly, PERK activation stimulates lipogenesis and gluconeogenesis by inducing the expression of CHOP through eukaryotic initiation factor 2 α (eIF2α)/ATF4 pathway [12]. Thirdly, ER stress induces the expression of the nuclear form of ATF6, which inhibits gluconeogenesis and lipogenesis by binding to and inactivating CREB-regulated transcription coactivator 2 (TORC2) and sterol-regulatory element binding protein 2 (SREBP2), respectively [13,14]. Lastly, ER stress stimulates the splicing of X-box binding protein 1 (Xbp1), which can directly or indirectly (through SREBP1) activate lipogenesis program while inhibiting gluconeogenesis [15,16]. ER stress is induced by unfolded protein response (UPR), which can be stimulated by some chemicals [3].

Tunicamycin, a pharmacological ER stress inducer, can stimulate tumor cell apoptosis [17,18]. Therefore, it has been used to develop an anti-cancer drug [19,20]. Because of its ER stress inducing character, tunicamycin has been reported to induce metabolism disorders in many studies. As is demonstrated in a study on hepatocytes, tunicamycin inhibits the phosphorylation of protein kinase B (Akt) [21], which plays a vital role in insulin sensitivity and in the regulation of glucose and triglyceride metabolism [22]. Wang et al. suggested that short-time tunicamycin treatment inhibited hepatic gluconeogenesis [13]. Furthermore, tunicamycin was revealed in a recent study to induce triglyceride accumulation in cultured HepG2 hepatic cells [23]. Chang and his colleagues demonstrated that short-term tunicamycin treatment increased hepatic lipid accumulation [24]. However, the in vivo effects of tunicamycin on hepatic gluconeogenesis, triglyceride accumulation and glycogen synthesis, and the exact mechanism have not been fully illustrated yet.

In the current study, the effects of 24 h tunicamycin treatment on serum lipids profiles, hepatic triglyceride accumulation, blood glucose level and liver glycogen content were explored using a mouse model. Meanwhile, the possible regulatory mechanism was investigated. Results indicated that, as compared with the control group, 24 h tunicamycin exposure dramatically increased hepatic triglyceride accumulation, inhibited liver lipoprotein secretion, decreased blood glucose level and hepatic glycogen content.

## 2. Results

### 2.1. Tunicamycin Induced Endoplasmic Reticulum Stress

**Figure 1:**
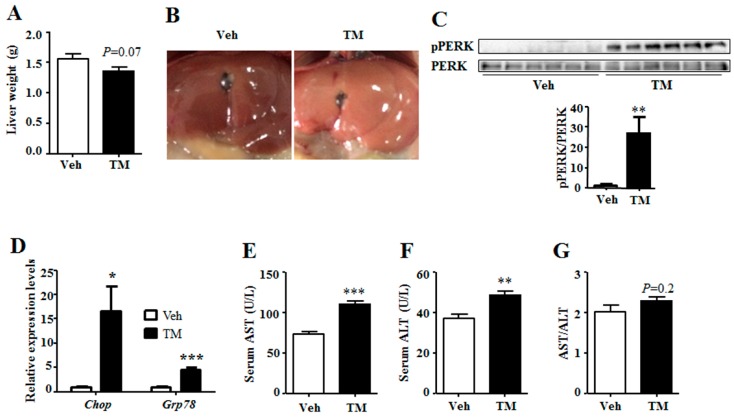
Tunicamycin induced hepatic endoplasmic reticulum (ER) stress and impaired liver function. 7-month-old mice were injected intraperitoneally with vehicle or 1 mg/kg tunicamycin. Livers were collected 24 h after injection under fed condition. *N* = 6 per group. (**A**) Liver weight at harvest; (**B**) Liver morphology; (**C**) Phosphorylation level of PERK in the liver; (**D**) Expression levels of ER stress indicator genes in the liver; (**E**) Serum level of AST; (**F**) Serum level of ALT; (**G**) The ratio of serum AST to ALT. Data were shown as mean ± SEM. Veh, vehicle; TM, tunicamycin. * *p* < 0.05, ** *p* < 0.01, *** *p* < 0.001 TM vs. Veh.

To investigate the effect of tunicamycin on liver energy homeostasis, mice were administrated with tunicamycin or vehicle for 24 h and were harvested under fed state. Result showed that tunicamycin did not alter the body weight when compared with the control group (Figure S1). However, the liver turned yellow, and the weight tended to decrease in tunicamycin-treated mice as compared to that of the control mice (Figure 1A,B). As tunicamycin is a pharmacologic ER stress inducer, the ER stress levels were detected in the liver. Results showed that, when compared with the control group, the phosphorylation level of PERK, an indicator of ER stress [3], was greatly induced in tunicamycin-treated liver (Figure 1C). In addition, tunicamycin up-regulated the expression of *Chop* and 78 kDa glucose-regulated protein (Grp78) (Figure 1D), two indicator genes of ER stress [25,26]. These data indicated that 24 h tunicamycin administration induced ER stress in the liver. In addition, the serum aspartate transaminase (AST) and alanine transaminase (ALT) levels were detected to reflect the liver function, which showed that the serum levels of both AST and ALT were higher in tunicamycin treated mice than these in the control mice (Figure 1E,F). However, the ratio of AST to ALT was unchanged (Figure 1G). These data indicated that tunicamycin slightly impaired liver function.

### 2.2. Tunicamycin Administration Decreased Blood Glucose Level

**Figure 2:**
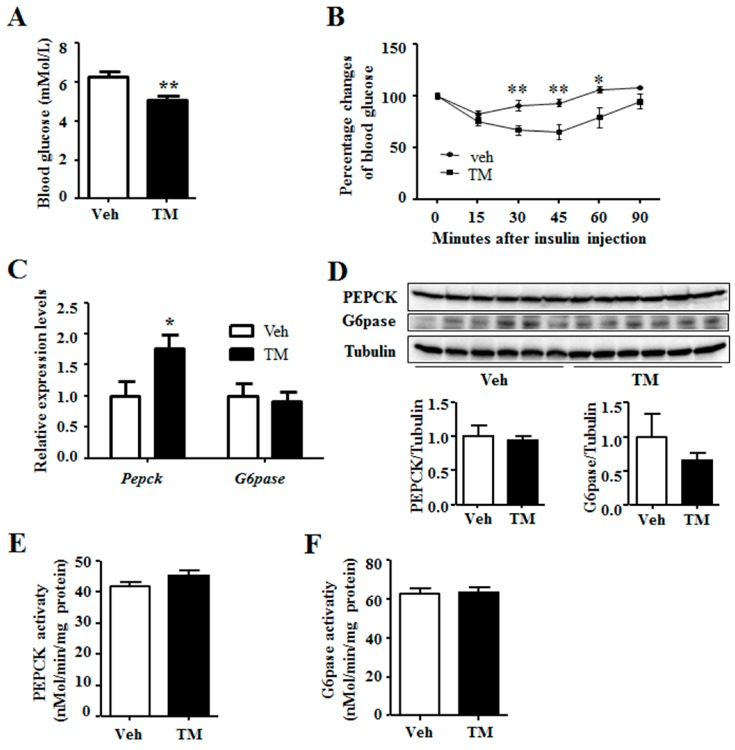
Tunicamycin reduced blood glucose level. (**A**) Blood glucose levels under fed state; *N* = 6 per group; (**B**) Insulin tolerance test. *N* = 7 per group; (**C**) Expression of gluconeogenic genes. *N* = 6 per group; (**D**) Western blotting bands and quantification for liver proteins; (**E**) The enzyme activity of PEPCK; (**F**) The enzyme activity of G6pase. Veh, vehcle; TM, tunicamycin. * *p* < 0.05, ** *p* < 0.01 TM vs. Veh.

It is reported that ER stress regulates hepatic gluconeogenesis [4], thus the blood glucose level was measured. Results indicated that, tunicamycin-treated mice had lower blood glucose levels as compared to the control mice (Figure 2A). In addition, insulin tolerance test revealed that tunicamycin-treated mice were more sensitivity to insulin than the control ones (Figure 2B). Nonetheless, tunicamycin did not affect the ability of glucose tolerance in mice (Figure S1B). In addition, serum insulin levels in tunicamycin-treated mice were similar to the control mice (Figure S1C). Gluconeogenesis plays an important role in systemic glucose homeostasis, while PEPCK and G6pase are the two rate-limiting enzymes for gluconeogenesis [2]. The results showed that tunicamycin up-regulated the expression of *Pepck* while *G6pase* expression remained unaffected (Figure 2C). Protein levels of PEPCK and G6Pase were also determined, which indicated that the two in tunicamycin-treated liver were not different from those in control liver (Figure 2D). Further study revealed no significant difference in the activity of PEPCK and G6Pase between tunicamycin group and control group (Figure 2E,F). Besides, liver content of pyruvate, the substrate of gluconeogenesis, trended to be higher in tunicamycin-treated mice than that in the control mice (Figure S1D). These findings demonstrated that 24 h tunicamycin treatment decreased blood glucose but it did not affect liver gluconeogenesis.

### 2.3. Tunicamycin Induced Hepatic Triglyceride Accumulation

**Figure 3:**
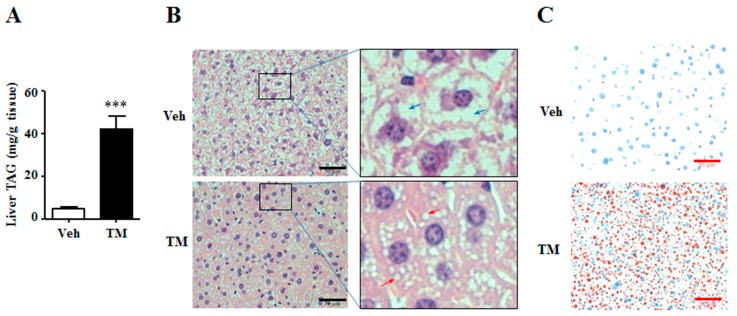
Tunicamycin induced liver triglyceride accumulation. 7-month-old mice were intraperitoneally injected with vehicle or 1 mg/kg tunicamycin. Livers were collected 24 h after injection. *N* = 6 per group. (**A**) Liver triglyceride content; (**B**) H&E staining of liver. Red arrows indicated lipid droplets, and blue arrows stood for glycogen particles; (**C**) Oil-red O staining of liver. Scale bars were equal to 50 μm. Data were expressed as mean ± SEM. Veh, vehicle; TM, tunicamycin. *** *p* < 0.001 TM vs. Veh.

The altered liver weight indicated that the content of tunicamycin-treated liver might be different from the content of control liver. Thus, the triglyceride and glycogen contents were detected in the liver. Results showed that tunicamycin remarkably increased the content of hepatic triglyceride (Figure 3A), an important energy source in the liver [27]. Meanwhile, H&E staining and Oil Red O staining also revealed that livers from tunicamycin treated mice had accumulated more and larger lipid droplets than those from control ones (Figure 3B,C). These data suggested that 24 h tunicamycin administration was susceptible to induce hepatic steatosis.

### 2.4. Tunicamycin Inhibited Hepatic Lipoprotein Secretion

**Figure 4:**
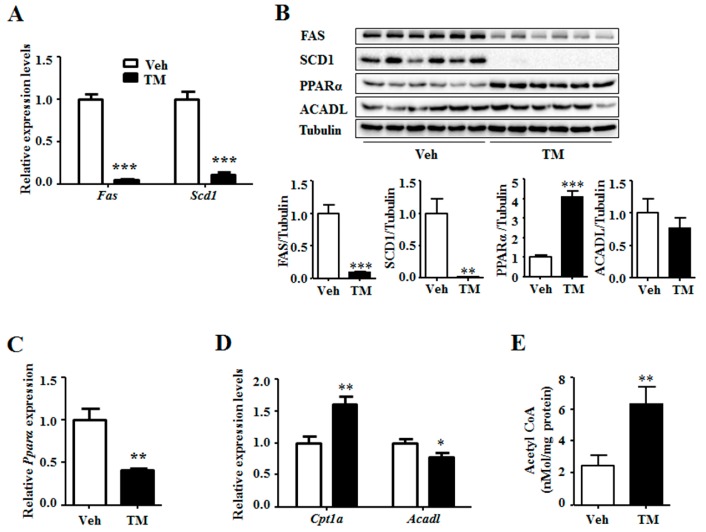
Tunicamycin inhibited the expression of liver lipogenic genes and increased the protein level of PPARα. (**A**) Expression of lipogenic genes; (**B**) Protein levels of lipogenic and fatty acid oxidation related genes; (**C**) Expression of fatty acid oxidation stimulator gene *Pparα*. (**D**) Expression of fatty acid oxidation related genes; (**E**) The acetyl CoA content in the liver. *N* = 6 per group. Veh, vehcle; TM, tunicamycin. * *p* < 0.05, ** *p* < 0.01, *** *p* < 0.001 TM vs. Veh.

ER stress has been reported to stimulate lipogenesis [4]. Thus, the expression levels of lipogenic genes were measured. The results indicated that, the expression of *Fas* and *Scd1* were notably down-regulated by tunicamycin rather than being up-regulated (Figure 4A). This result was supported by the decreased protein levels of FAS and SCD1 in the tunicamycin treated liver (Figure 4B). Consequently, the expression of fatty acid oxidation related genes was thus detected. Results showed that tunicamycin inhibited the gene expression of *Pparα*, a transcription stimulator of fatty acid oxidation genes (Figure 4C). However, the protein level of PPARα was significantly increased in tunicamycin-treated liver as compared to that in the control liver (Figure 4B). Meanwhile, the gene expression level of *Cpt1a*, an important fatty acid oxidation related gene, was increased in the tunicamycin-treated liver (Figure 4D). Furthermore, though the gene expression level of *Acadl*, another fatty acid oxidation gene, was decreased in tunicamycin-treated liver as compared to that in the control liver, the protein level was unchanged (Figure 4B,D). Besides, the content of acetyl CoA, the substrate for lipogenesis and production of fatty acid oxidation, was higher in tunicamycin-treated liver than this in the control liver (Figure 4E). These data suggested that the increased triglyceride content in 24 h tunicamycin-treated liver was probably independent on lipogenesis and fatty acid oxidation.

**Figure 5:**
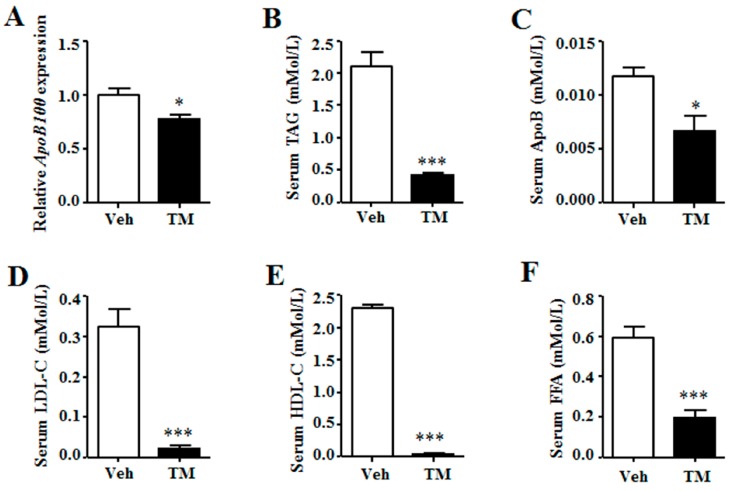
Tunicamycin impaired hepatic lipoprotein secretion. (**A**) Gene expression of *ApoB100* in the liver; (**B**) Serum levels of triglyceride; (**C**) Serum levels of apolipoprotein B; (**D**) Serum levels of low-density lipoprotein cholesterol; (**E**) Serum levels of high-density lipoprotein cholesterol; (**F**) Serum levels of free fatty acid. *N* = 6 per group. Veh, vehcle; TM, tunicamycin. * *p* < 0.05, *** *p* < 0.001 TM vs. Veh.

The lipoprotein secretion of liver was then analyzed. Data indicated that the expression of *ApoB100*, the major apolipoprotein of liver, was inhibited in tunicamycin-treated liver as compared to that in the control liver (Figure 5A). Meanwhile, serum levels of triglyceride, ApoB, low-density lipoprotein cholesterol (LDL-C) and high-density protein cholesterol (HDL-C) were significantly lower in tunicamycin-treated mice than those in control mice (Figure 5B–E). Besides, serum free fatty acid (FFA) level was also decreased in tunicamycin-treated mice (Figure 5F). These data indicated that tunicamycin impaired hepatic lipoprotein secretion.

### 2.5. Tunicamycin Attenuated Liver Glycogen Accumulation

The results showed that tunicamycin had decreased the content of liver glycogen (Figure 5A), another important energy source in liver besides triglyceride [10]. Morphological analysis of H&E and PAS staining also led to similar result (Figure 2B and Figure 5B). Akt activation stimulates liver glycogen synthesis [28]. Therefore, the phosphorylation level of Akt was investigated, the results of which showed that tunicamycin reduced the phosphorylation level of Akt (Figure 5C). However, tunicamycin did not alter the phosphorylation level or the total protein level of GS (Figure 5C), neither the protein level of GP (Figure 5C). These data suggested that glycogen content might be reduced in a GS- and GP-expression-independent manner in 24 h tunicamycin-treated liver.

## 3. Discussion

ER stress has dual functions, as it induces metabolic syndrome as well as inducing tumor cell apoptosis [3]. As is shown in this study, 24 h tunicamycin treatment notably induces hepatic ER stress and yellowish color, impairs liver function, reduces blood glucose level, stimulates hepatic triglyceride accumulation and attenuates liver glycogen storage. However, it is also observed that 24 h tunicamycin treatment has no effect on the expression and the activity of gluconeogenic enzymes, which disagrees with previous report, who indicates that tunicamycin inhibits the expression of *Pepck* and *G6pase*. Besides, we observe that, though the gene expression is inhibited, the protein level of PPARα is significantly increased in tunicamycin treated liver, which has not been reported previously. In addition, our study demonstrates that tunicamycin decreases hepatic lipoprotein secretion by inhibiting the expression of ApoB100, which has not been reported previously either. Our study also demonstrates that the increased hepatic triglyceride accumulation may be due to the inhibited hepatic lipoprotein secretion.

### 3.1. Tunicamycin and ER Stress

Results in this study have shown that tunicamycin exposure has greatly induced the expression of *Chop* and *Grp78* and the phosphorylation level of PERK (Figure 1), suggesting that 24 h tunicamycin treatment gives rise to remarkable ER stress. ER stress has a dominant effect on metabolic homeostasis, especially for chronic ER stress, which accounts for one of the reasons for chronic metabolic diseases [3,4,29]. Three types of ER stress have been reported, including acute ER stress, periodic ER stress and chronic ER stress, and different types of ER stress have different effects on metabolism [4]. Of them, the effects of acute and chronic ER stress on hepatic energy metabolism have been widely studied [3,4,30]. However, the effect of sub-acute ER stress, the transition between acute and chronic ER stress, on metabolic homeostasis remains unknown. Acute ER stress has been studied within hours while chronic ER stress within days or longer; therefore, ER stress induced by 24 h tunicamycin treatment in our study is considered as the transition between acute and chronic ER stress [3,4,30]. This may account for the reason why the effect of tunicamycin on the expression of hepatic gluconeogenic genes in our study is different from those in previous reports. Consequently, one possibility has been raised in this study that the sub-acute ER stress has different effects on hepatic energy metabolism from those of the acute and chronic ER stress. Further studies are required to explore the role of sub-acute ER stress on energy metabolism.

Besides, tunicamycin is reported to inhibit tumor growth at dose of 1 mg/kg, but not at the dose of 0.5 mg/kg [19]. However, our study indicates that tunicamycin at the dose of 1 mg/kg body weight slightly impairs liver function. This suggests that when tunicamycin or other ER stress inducers are used as anti-cancer medicine, liver protection is required.

### 3.2. Tunicamycin and Blood Glucose

It is shown in the current study that 24 h tunicamycin treatment decreases the blood glucose level (Figure 4), which is supported by previous reports that acute tunicamycin treatment reduces blood glucose level [13,31]. However, 24 h tunicamycin treatment makes no difference to the expression of gluconeogenic genes in this study, which is different from the previous reports declaring an inhibition of gluconeogenic genes expression by acute tunicamycin treatment. One possible reason is the duration of tunicamycin treatment, which is 24 h in this study, compared to 10 h and 6 h in the studies by Wang et al. and Lee et al., respectively [13,31]. Moreover, our result is also different from the report demonstrating that chronic ER stress induced by long term high fat diet intake stimulates gluconeogenesis [32].

Thus, another possibility has been raised that tunicamycin induces apoptosis of some liver cells. This may lead to decreased hepatic glucose production rate in tunicamycin-treated mice. Thus, when challenged with insulin, blood glucose levels of the tunicamycin-treated mice decrease faster than these of the control mice. At the same time, the expression and activity of PEPCK and G6pase are normalized to total β-action (gene expression), total tubulin (protein expression) or total protein (enzyme activity), including which of the apoptosis cells. So, they retain similar in both groups or is higher (gene expression of *Pepck*) in the tunicamycin treated liver. However, the exact mechanism needs further study to be elucidated.

### 3.3. Tunicamycin and Hepatic Triglyceride

In this study, tunicamycin is found to stimulate liver triglyceride accumulation (Figure 1), which is consistent with the previous studies by Chang et al. and by Lee et al. In their study, Chang et al. reported that tunicamycin induced triglyceride accumulation in hepatocytes, while Lee et al. observed that tunicamycin increased the triglyceride content in mouse liver [24,30]. Increased hepatic triglyceride accumulation induces hepatic steatosis [8,27,33]. Thus, it is suggested from the findings of this study that prolonged tunicamycin treatment may lead to increased risk of hepatic steatosis.

Triglyceride metabolism in liver is regulated by lipogenesis and fatty acid β-oxidation [27]. Chronic ER stress is reported to induce lipogenesis in the liver [4]. However, 24 h tunicamycin treatment down-regulates the gene expression and protein levels of lipogenic enzymes FAS and SCD1, which are important lipogenic genes (Figure 2). This may be attributed to the sub-acute ER stress induced by 24 h tunicamycin exposure in our study, which has a different effect on lipogenesis from that of chronic ER stress [4]. Lee et al. declared an increased lipogenesis in tunicamycin-treated liver. However, the expression of *Fas* and *Scd1* in the cultured hepatoma cells, rather than in the liver, was shown in their study [30]. The decreased expression of lipogenic genes in 24 h tunicamycin-treated liver may result from the negative feedback of increased hepatic triglyceride accumulation on lipogenesis [7]. Akt activation is reported to stimulate hepatic lipogenesis [34]. Therefore, decreased Akt phosphorylation level may also be responsible for the inhibition of lipogenic gene expression by 24 h tunicamycin exposure in our study (Figure 3). However, more studies are required to further elucidate the precise mechanism by which tunicamycin down-regulates the expression of lipogenic genes.

Subsequently, the expression levels of fatty acid oxidation related genes are detected. The result shows that, the expression of *Pparα* is significantly decreased, which is in accordance with a previous report [30]. However, the protein level of PPARα is significantly higher in tunicamycin-treated liver than that in the control liver. This suggests that tunicamycin has contrary effects on transcriptional and post-transcriptional expression of PPARα. However, the mechanism and the role need further study to be explored. In addition, the expression of *Cpt1a* is increased and the protein level of ACADL was unchanged by tunicamycin. Moreover, liver content of acetyl CoA, the substrate for lipogenesis and production of fatty acid oxidation, is increased in tunicamycin treated mice. All these data demonstrate that the fatty acid oxidation is probably not decreased by tunicamycin in our study.

Liver triglyceride content also can be regulated by hepatic lipoprotein secretion [35]. Our data demonstrate that the expression of *ApoB100*, the liver expressed VLDL binding apolipoprotein, is reduced in the tunicamycin treated liver. Together with the reduction of serum triglyceride, ApoB, LDL-C and HDL-C levels, our data suggest that the hepatic lipoprotein secretion of tunicamycin-treated mice is inhibited, which may be the reason why hepatic triglyceride accumulation is increased in tunicamycin-treated mice.

### 3.4. Tunicamycin and Liver Glycogen

**Figure 6:**
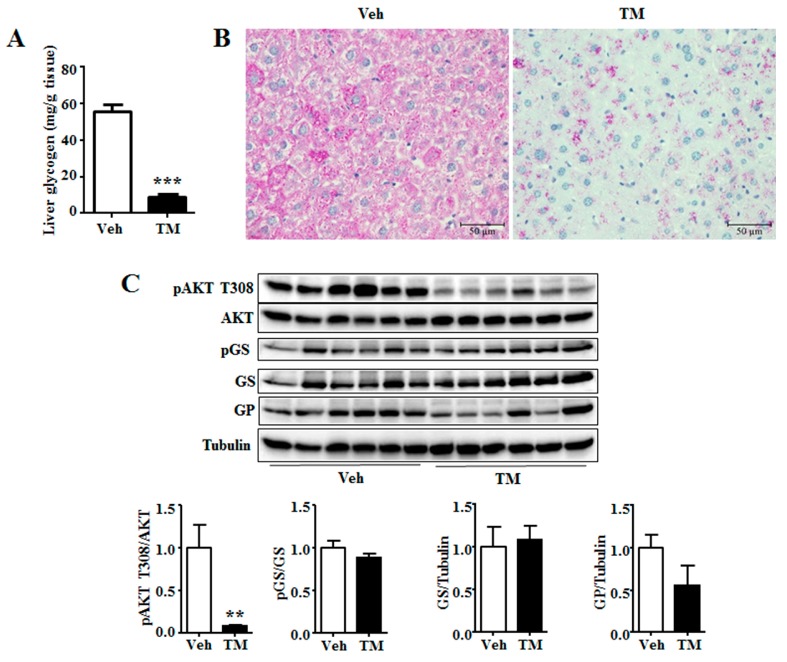
Tunicamycin decreased hepatic glycogen content. (**A**) Hepatic glycogen content; (**B**) PAS staining of liver; (**C**) Western blotting bands and quantification of liver proteins. *N* = 6 per group. Scale bars were equal to 50 μm. Veh, vehcle; TM, tunicamycin. ** *p* < 0.01, *** *p* < 0.001 TM vs. Veh.

Hepatic glycogen is a storage form as well as a rapid source of blood glucose, while decreased hepatic glycogen storage adds to the risk of hypoglycemia under fasting condition [36]. 24 h tunicamycin treatment evidently decreases hepatic glycogen content in our study (Figure 6). It suggests that tunicamycin has impaired liver glycogen accumulation, which has not been reported previously. Akt is reported to stimulate hepatic glycogen synthesis by means of stimulating GS activity [28]. A decrease of Akt phosphorylation level has been observed in this study (Figure 3). However, the phosphorylation level or the total protein level of GS remains unchanged in tunicamycin-treated mice, indicating that tunicamycin may decrease liver glycogen content in a GS-expression independent manner. Besides, tunicamycin does not change the protein level of GP as well (Figure 3), suggesting that glycogen breakdown may not be affected either. Therefore, the reduced hepatic glycogen content may result from the decreased blood glucose level in tunicamycin-treated mice. These results indicate that cancer patients may be fasting intolerance during the treatment of tunicamycin or other ER stress inducing medicines.

Overall, 24 h tunicamycin exposure slightly impairs liver function, increases hepatic triglyceride accumulation, inhibits liver lipoprotein secretion and decreases blood glucose level, hepatic glycogen accumulation and serum lipids levels. Our study suggests that, chemicals, which are used to treat tumors by their ER stress inducing characteristic, may cause liver function failure and metabolic disorders, thus liver protection should be performed when these medicines are using.

## 4. Materials and Methods

### 4.1. Animals and Chemicals

Animal study protocol (MICE2015016, 10/22/2016) was reviewed and approved by the Animal Care and Use Committee of Sichuan Agricultural University. All animal procedures were performed according to the National Institutes of Health guide for the care and use of Laboratory animals. 7-month-old C57BL/6 male mice were obtained from Vital River Laboratory Animal Technology Co. Ltd. (Beijing, China). The mice were allowed for one week of acclimation in a pathogen-free room at the temperature of 22 °C and the humidity of 60%. Subsequently, they were randomly divided into 2 groups according to similar average body weight and blood glucose level (Blood glucose strips were purchased from Beijingyicheng, Beijing, China). One group were injected intraperitoneally (IP) with 1 mg/kg tunicamycin (Sigma, Shanghai, China) or vehicle. The body weight and blood glucose level at fed state were measured 24 h after injection. Later, mice were euthanized using carbon dioxide, followed by cervical dislocation. Serum was collected for further analysis. Livers were rapidly dissected and weighed after taking morphological images. One piece of liver was fixed in 10% formalin for H&E and PAS staining, while one piece was frozen in OCT embedding medium, and the remaining was frozen at −80 °C for further analysis.

### 4.2. Insulin Tolerance Test and Glucose Tolerance Test

For insulin tolerance test (ITT), mice were injected intraperitoneally with 1 mg/kg tunicamycin or vehicle. 18 h later, mice were deprived of food for 6 h. And then, mice were injected intraperitoneally with 0.25 U/kg insulin (Novo Nordisk, Beijing, China). Blood glucose level was measured 0, 15, 30, 45, 60 and 90 min after insulin injection.

For glucose tolerance test (GTT), mice were intraperitoneally injected with vehicle or 1 mg/kg tunicamycin, and then were deprived of food 8 h after injection. 24 h after injection, mice were intraperitoneally injected with 1 g/kg dextrose (Sigma). Blood glucose were measured at 0, 15, 30, 45, 60 and 90 min after dextrose injection.

### 4.3. Serum Metabolites Profile Analysis

Serum AST, ALT, triglyceride, ApoB, HDL-C, LDL-C and FFA levels were measured on an automatic biochemical analyzer (7020, HITACHI, Tokyo, Japan) with their analysis kits respectively (All kits were obtained from Makerbio. Co., Chengdu, China) according to the manufacturer’s instructions. Serum pyruvate levels were detected using a commercial kit (Nanjing Jiancheng Bioengineering Institute, Nanjing, China) according to the manufacturer’s instruction.

### 4.4. Serum Insulin Level Measurement

Serum insulin levels were measured with a mouse insulin ultrasensitive ELISA kit (ALPCO, Salem, MA, USA) according to the manufacturer’s instruction.

### 4.5. Liver Pyruvate and Acetyl CoA Content Measurement

Liver pyruvate content was detected using a commercial pyruvate kit (Nanjing Jiancheng Bioengineering Institute, Nanjing, China) according to the manufacturer’s instruction. Liver acetyl CoA content was detected using a commercial acetyl CoA kit (Cominbio, Suzhou, China) according to the manufacturer’s instruction.

### 4.6. Liver Glycogen Assay

Liver glycogen content was measured using a commercial glycogen assay kit from Nanjing Jiancheng Bioengineering Institute according to the manufacturer’s instruction with slight modification. Briefly, frozen livers were ground with a pestle in liquid nitrogen. 300 µL alkaline solution was added to 100 mg tissue powder. Mixture was heated in boiling water for 20 min. Samples were then mixed with 96-fold (volume/tissue weight) of doubly distilled (dd) H_2_O and centrifuged at 5000× *g* for 5 min. 200 μL of the supernatant or standard was transferred to a new Eppendorf (EP) tube and mixed with 400 μL chromogenic agent. The mixture was subsequently heated at 98 °C for 5 min. The absorbance was measured at 620 nm on a plate reader (SpectraMax M2, Molecular Devices, Sunnyvale, CA, USA). Liver glycogen content was calculated from the known standards multiplied by the dilution factor.

### 4.7. Liver Triglycerides Assay

Liver triglyceride content was measured using a commercial triglyceride assay kit from Nanjing Jiancheng Bioengineering Institute according to the manufacturer’s instruction with modifications as previously described [37]. Briefly, 50 mg tissue powder was homogenized in 1.5 mL pure ethanol with a PowerGen 125 homogenizer (Thermo Fisher Scientific, Waltham, MA, USA). The homogenate was vortexed, and then was centrifuged at 12,000× *g* for 5 min. 1 mL of the supernatant was transferred to a new EP tube. 5 μL samples and standards (Cat# 17811-1AMP, Sigma) were added into a 96-well transparent plate and were incubated with 200 μL enzyme mixture at 37 °C for 10 min. The absorbance was measured at 510 nm on a plate reader (Spectra Max M2). Liver triglycerides content was calculated from the known standards multiplied by the dilution factor.

### 4.8. PEPCK and G6pase Activity Analysis

The activities of hepatic PEPCK and G6pase were measured using their activity kits respectively, according to the manufacturer’s instruction (Cominbio, Suzhou, China).

### 4.9. Liver Histology Staining

For H&E and PAS staining, fresh liver tissues were fixed in 10% formalin for 48 h, and then dehydrated and embedded in paraffin. Embedded tissues were sliced into 4 μm sections (RM2016, Leica, Shanghai, China). For H&E staining, the sections were dehydrated, stained with hematoxylin for 5 min, washed with ddH_2_O, and stained with eosin for 2 min. The sections were then dehydrated and mounted with a neutral resin onto slides. For PAS staining, the sections were dehydrated, incubated with 0.5% periodate solution for 10 min, washed with ddH_2_O, stained with Schiff solution for 30 min in a dark room, washed with ddH_2_O, and then stained with hematoxylin for 2 min. The sections were washed with ddH_2_O, dehydrated, and mounted with a neutral resin onto slides.

For Oil Red O staining, fresh liver tissues were embedded in OCT embedding medium and frozen in liquid nitrogen, and then sliced into 10 μm sections on a frozen section machine (Cryotome E, Thermo Fisher Scientific). The sections were dried on slides, fixed in 4% paraformaldehyde for 15 min, washed with PBS, stained with Oil Red O staining solution for 1 h, washed again with PBS, and stained with hematoxylin for 2 min. The slides were then washed with ddH_2_O, dried, and mounted with glycerogelatin. Images were captured on a microscope (TS100, Nikon, Tokyo, Japan) with a CCD (DS-U3, Nikon) using imaging software (NIS-Elements F3.2, Nikon).

### 4.10. RNA Extraction and Real-Time PCR

RNA extraction and real-time PCR were performed as previously reported [37]. Briefly, 50 mg liver tissue powder was homogenized in 1 mL Trizol Reagent (Invitrogen, Shanghai, China) and RNA was extracted in accordance with the manufacturer’s instruction. The quality of RNA was assessed by agarose gel and the concentration was measured with a spectrophotometer (NanoDrop 2000, Thermo Fisher Scientific). 1 μg RNA was reverse-transcribed into cDNA with a reverse-transcription PCR kit according to the manufacturer’s instructions (Takara, Dalian, China). Real-time PCR was conducted on a quantitative-PCR machine (7900HT, ABI, Carlsbad, CA, USA) with Power SYBR Green RT-PCR reagents (Thermo Fisher Scientific). The following reagent amounts were used for each reaction: forward primer, 300 nM; reverse primer, 300 nM; cDNA sample, 20 ng. The conditions used for PCR were: 95 °C for 10 min for 1 cycle, and then 40 cycles of 95 °C for 15 s followed by 60 °C for 1 min. The real time PCR data was analyzed by the 2^−ΔΔ*C*t^ method with *β-actin* as the reference. The sequences of the primers are listed below:

*β-actin* forward GGCTGTATTCCCCTCCATCG and reverse CCAGTTGGTAACAATGCCATGT; *Chop*, forward CACGCACATCCCAAAGCC and reverse GGGCACTGACCACTCTGTT; *Grp78*, forward ATCAGGGCAACCGCATCAC and reverse TGATGTCCTGCTGCACCGAA; *Pepck*, forward CGCTGGATGTCGGAAGAGG and reverse GGCGAGTCTGTCAGTTCAATAC; *G6pase*, forward CGACTCGCTATCTCCAAGTGA and reverse GTTGAACCAGTCTCCGACCA; *Fas*, forward GGCTCTATGGATTACCCAAGC and reverse CCAGTGTTCGTTCCTCGGA; *Scd1*, forward CCTACGACAAGAACATTCAATCCC and reverse CAGGAACTCAGAAGCCCAAAGC; *Pparα*, forward TACTGCCGTTTTCACAAGTGC and reverse AGGTCGTGTTCACAGGTAAGA; *Cpt1a*, forward CTCCGCCTGAGCCATGAAG and reverse CACCAGTGATGATGCCATTCT; *Acadl*, forward TCTTTTCCTCGGAGCATGACA and reverse GACCTCTCTACTCACTTCTCCAG; *ApoB100*, forward TTGGCAAACTGCATAGCATCC and reverse TCAAATTGGGACTCTCCTTTAGC.

### 4.11. Western Blot Analysis

Western blot analysis was performed as previously reported [38]. For the preparation of protein lysates, 100 mg liver tissue powder was homogenized in 1 mL cell lysis buffer (Beyotime Biotechnology, Shanghai, China) supplemented with protease inhibitor cocktail (Roche, Mannheim, Germany) on a homogenizer. The protein lysate was centrifuged at 12,000× *g* and 4 °C for 30 min, and the supernatant was transferred to a new EP tube. The concentration of protein in the supernatant was measured with a BCA Protein Assay Kit (Thermo Fisher Scientific). 100 μg protein was used to prepare an electrophoresis sample with loading buffer (BioRad, Shanghai, China) in a volume of 30 μL for each sample. Proteins were separated on 12% polyacrylamide gel, and then transferred onto PVDF membranes (BioRad). The membranes were blocked in 1% BSA/1× TBST for 1 h at room temperature, followed by incubation with the appropriate primary antibodies (1 ug/ml) overnight. ACADL antibody was the product of Abcam (Shanghai, China); pAKT T308 (4056), AKT (9272), pGS (3891), GS (3886), pPERK (3179), PERK (5683), SCD1 (2794) and tubulin (3873) antibodies were obtained from Cell Signaling Technology (Shanghai, China); and GP (sc-46347), G6pase (sc-398155), PEPCK (sc-377136), FAS (sc-48357) and PPARα (sc-398394) antibodies were obtained from Santa Cruz (Shanghai, China). After thorough washing, membranes were incubated with appropriate horseradish peroxidase-linked secondary antibodies (7074 and 7076, CST) (1:2000 dilution in 5% milk/1× TBST) for 1 h. After further thorough washing, protein signals were detected by ECL western blotting detection reagent (BioRad, Shanghai, China) on a Molecular Imager ChemiDoc XRS+ System (BioRad, Shanghai, China). Blots were quantified with ImageJ software (National Institutes of Health, Bethesda, MD, USA).

### 4.12. Statistical Analysis

Data were analyzed with Microsoft Office Excel 2016 and Prism 5, and the results were presented as mean ± SEM. Student’s *t*-test was used to compare the difference between two groups. Statistical significance was determined at *p* < 0.05.

## Supplementary Materials

Supplementary materials can be found at www.mdpi.com/1422-0067/18/8/1710/s1.

## Abbreviations

TM	Tunicamycin
ER	Endoplasmic reticulum
GRP78	Glucose-regulated protein 78
CHOP	CCAAT/enhancer-binding protein (C/EBP) homologous protein
PERK	Protein kinase R-like endoplasmic reticulum kinase
AST	Aspartate transaminase
ALT	Alanine transaminase
ApoB100	Apolipoprotein B100
ApoB	Apolipoprotein B
LDL-C	Low-density lipoprotein cholesterol
HDL-C	High-density lipoprotein cholesterol
FFA	Free fatty acid
Akt	Protein kinase B
GS	Glycogen synthetase
GP	Glycogen phosphotase
FAS	Fatty acid synthase
SCD1	Stearoyl-CoA desaturase 1
PPARα	Peroxisome proliferator-activated receptor alpha
CPT1a	Carnitine palmitoyltransferase 1a
ACADL	Acyl-CoA dehydrogenase, long chain
PEPCK	Phosphoenolpyruvate carboxykinase
G6pase	Glucose 6-phosphatase
