# Supplementary material for: Endoplasmic Reticulum Stress Inducer Tunicamycin Alters Hepatic Energy Homeostasis in Mice
Source: Int J Mol Sci. 2017 Aug 4;18(8):1710. doi: 10.3390/ijms18081710 (PMC5578100; doi:10.3390/ijms18081710)
Supplement: Supplementary file 1 [file ijms-18-01710-s001.pdf]

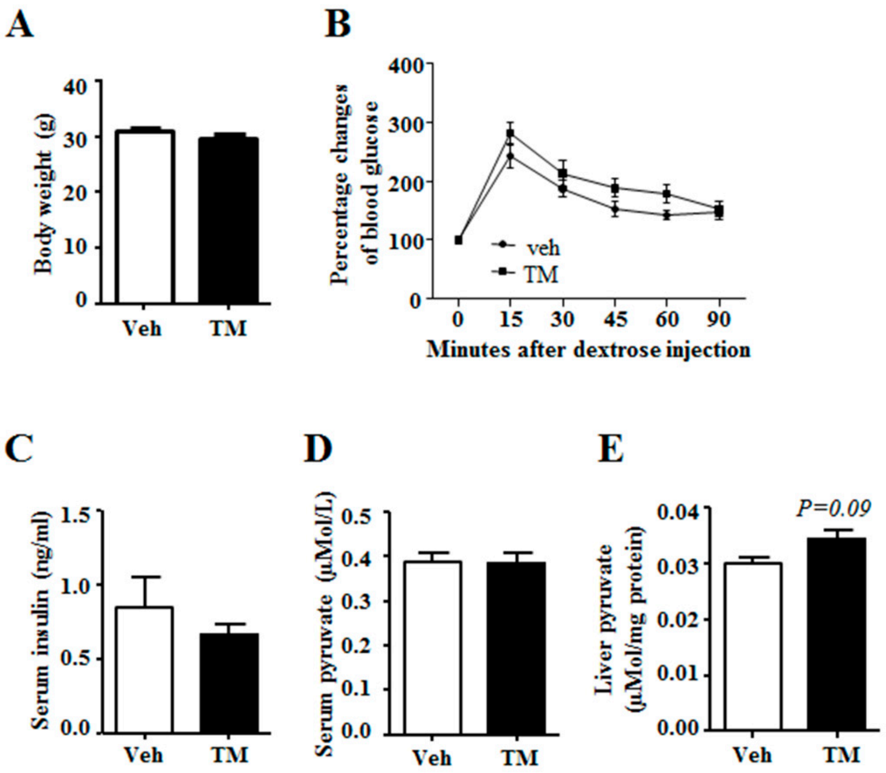

Figure S1. The effect of tunicamycin on body weight, GTT and serum insulin level. 7-month-old mice were intraperitoneally injected with vehicle or 1 mg/kg tunicamycin. Serum and livers were collected 24 h after injection under fed condition. N=6 per group. (**A**) Body weight at harvest. (**B**) Glucose tolerance test. 7-month-old mice were intraperitoneally injected with vehicle or 1 mg/kg tunicamycin, and then were deprived of food 8 h after injection. 24 h after injection, mice were intraperitoneally injected with 1 g/kg dextrose. Blood glucose were measured at 0, 15, 30, 45, 60 and 90 min after dextrose injection. N=6 per group. (**C**) Serum insulin levels at harvest. (**D**) Serum pyruvate levels at harvest. (**E**) Liver pyruvate content. Data were shown as mean ± SEM. Veh, vehicle; TM, tunicamycin.
